# miR-370-3p Regulates Adipogenesis through Targeting Mknk1

**DOI:** 10.3390/molecules26226926

**Published:** 2021-11-17

**Authors:** Peiwen Zhang, Xinrong Li, Shunhua Zhang, Shuang Wu, Qian Xiao, Yang Gu, Xinyu Guo, Xutao Lin, Lei Chen, Ye Zhao, Lili Niu, Guoqing Tang, Yanzhi Jiang, Linyuan Shen, Li Zhu

**Affiliations:** 1 College of Animal Science and Technology, Sichuan Agricultural University, Chengdu 611130, China; zpw1995@stu.sicau.edu.cn (P.Z.); lixinrong0807@163.com (X.L.); zhangsh1919@163.com (S.Z.); wushuang@stu.sicau.edu.cn (S.W.); xiaoqian5022@163.com (Q.X.); guyang@stu.sicau.edu.cn (Y.G.); guoxinyu@stu.sicau.edu.cn (X.G.); lxt1796014680@163.com (X.L.); chenlei815918@163.com (L.C.); zhye3@foxmail.com (Y.Z.); dky9829@126.com (L.N.); tyq003@163.com (G.T.); jiangyz04@163.com (Y.J.); 2 Farm Animal Genetic Resources Exploration and Innovation Key Laboratory of Sichuan Province, Sichuan Agricultural University, Chengdu 611130, China

**Keywords:** miR-370-3p, adipogenesis, proliferation, differentiation, fatty acid composition

## Abstract

Excessive fat accumulation can lead to obesity, diabetes, hyperlipidemia, atherosclerosis, and other diseases. MicroRNAs are a class of microRNAs that regulate gene expression and are highly conserved in function among species. microRNAs have been shown to act as regulatory factors to inhibit fat accumulation in the body. We found that miR-370-3p was expressed at lower levels in the fat mass of mice on a high-fat diet than in mice on a normal control diet. Furthermore, our data showed that the overexpression of miR-370-3p significantly suppressed the mRNA expression levels of adipogenic markers. Thus, miR-370-3p overexpression reduced lipid accumulation. Conversely, the inhibition of miR-370-3p suppressed 3T3-L1 preadipocyte proliferation and promoted preadipocyte differentiation. In addition, Mknk1, a target gene of miR-370-3p, plays an opposing role in preadipocyte proliferation and differentiation. Moreover, consistent results from in vitro as well as in vivo experiments suggest that the inhibition of fat accumulation by miR-370-3p may result from the inhibition of saturated fatty acids that promote the accumulation of polyunsaturated fatty acids. In conclusion, these results suggest that miR-370-3p plays an important role in adipogenesis and fatty acid metabolism through the regulation of Mknk1.

## 1. Introduction

Obesity is a major global public health problem [1] and is closely linked with hypertension, cardiovascular disease [2], type 2 diabetes, etc. Obesity results from energy intake exceeding expenditure [3]. The adipose development process involves preadipocyte proliferation and adipocytes differentiation [4]. Adipogenesis is a crucial process that participates in adipocyte development and fat metabolism [5,6]. Hence, it is necessary to determine the adipogenesis mechanisms that are closely linked with severe diseases. In addition, a high number of studies have demonstrated that adipogenesis is tightly regulated by transcriptional and posttranscriptional regulation factors, and complex signaling pathways.

MicroRNA (miRNA) is a class of small RNA, which is approximately 22 nucleotides long and has no, or a very limited, protein-coding capacity [7]. Furthermore, miRNAs directly target and bind the 3′untranslated region (UTR) of mRNA, incorporating it into the RNA-induced silencing complex (RISC) [8]. Due to this property, miRNA regulates gene expression in a variety of biological processes. For instance, miR-9 inhibits 3T3-L1 cell adipogenesis by targeting Patatin-like phospholipase domain containing 3 (PNPLA3) [9]. In contrast, miR-17-5p and miR-106a promoted 3T3-L1 preadipocyte differentiation by repressing bone morphogenetic protein 2 (BMP2) [10]. However, the detailed mechanisms of miR-370-3p regulate preadipocyte proliferation and differentiation is unclear. In addition, MAP kinase-interacting serine/threonine kinase 1 (Mknk1) has shown that knockout Mknk1 in mice could prevent against the metabolic syndrome. Previous studies have only shown that the Mknk1 function in vitro, knockdown the Mknk1, which can regulate adipogenesis. Previous studies have shown that knockdown of Mknk1 in mice can improve insulin resistance in mice with obesity metabolic syndrome and can promote glucose metabolism in mice. It has also been shown that simultaneous knockout of both members of MNK is effective against obesity metabolic syndrome [11,12]. In addition, some bioinformatics studies have also suggested that Mknk1 may be associated with metabolic diseases such as polycystic ovary syndrome [13]. Most studies on the two members of the MNK family have focused on the proliferation and differentiation of Mknk2 in adipocytes and in vivo experiments, while few studies have been reported on the proliferation and differentiation of Mknk1 in adipocytes, and so forth.

Here, we have studied the critical role of miR-370-3p and Mknk1 in adipogenesis and revealed the underlying mechanism by which miR-370-3p promoted 3T3-L1 adipocyte proliferation and inhibited its differentiation. Furthermore, our data demonstrate that the mature adipocytes regulate fatty acid composition via directly targeting Mknk1.

## 2. Results

### 2.1. miR-370-3p Is Associated with Adipogenesis

**Figure 1:**
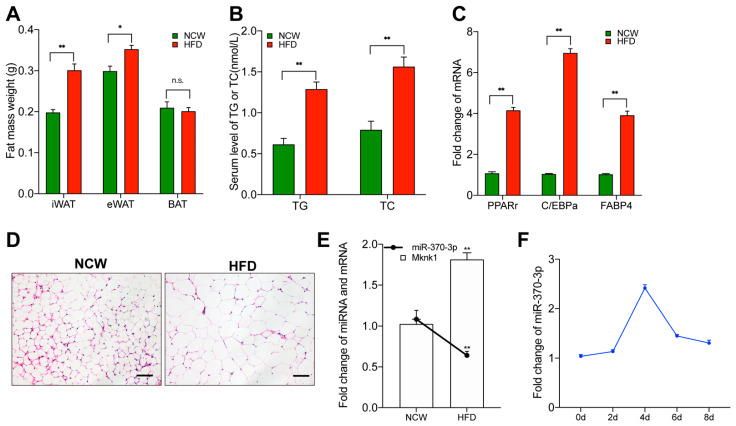
miR-370-3p is associated with adipogenesis. (**A**) The weight of iWAT, eWAT, BAT fat mass from HFD and NCW fed mice. (**B**) Triglycerides (TG) and total cholesterol (TC) were measured. (**C**) The mRNAs expression level of PPARγ, C/EBPa, FABP4 was measured in adipose tissues. (**D**) H&E staining for epididymal white adipose tissue (eWAT) from mice fed with HFD and NCW. (**E**) The expression of miR-370-3p and Mknk1 in eWAT from HFD- and NCW-fed mice. (**F**) The mRNAs expression level of miR-370-3p was quantified during the differentiation process. Scale bars were 50 μm. All results are presented as means ± SEM. n = 3 per treatment. Each treatment represents the mean of three replicates. * *p* < 0.05; ** *p* < 0.01, n.s. no significant difference.

As shown in Figure 1A, after a 6 week, high-fat diet, we found the obese mice model’s fat mass to be significantly heavier. The high-fat diet (HFD) mice had both increased epididymal white adipose tissue (eWAT) and inguinal white adipose tissue (iWAT) fat weight. The results revealed that the modeling was successful. In addition, as shown in Figure 1B,C, compared with the normal control diet group, the serum level of triglycerides (TG) and total cholesterol (TC) and the expression level of adipogenesis marker genes in eWAT, such as Peroxisome proliferator activated receptor γ (PPARγ), CCAAT/enhancer binding protein α (C/EBPα), and adipocyte fatty acid-binding protein 4 (FABP4), were significantly increased in the HFD group. As shown in Figure 1D, consistent with the previous study, the adipocyte area was significantly expanded in the high-fat diet group. Interestingly, miR-370-3p expression in eWAT was downregulated in the high-fat-diet group (Figure 1E). Mouse 3T3-L1 preadipocytes were always used as a characterized model for adipogenesis research. We treated 3T3-L1 preadipocyte with proliferation and differentiation phase and collected the cell sample to test the miR-370-3p expression during this process. We found that the miR-370-3p expression was increased during proliferation and cell differentiation prophase, while the expression declined in the late adipocyte differentiation stage (Figure 1F). These results indicated that miR-370-3p may participate in adipogenesis and play a critical role in preadipocyte differentiation.

### 2.2. miR-370-3p Promotes Preadipocyte Proliferation

**Figure 2:**
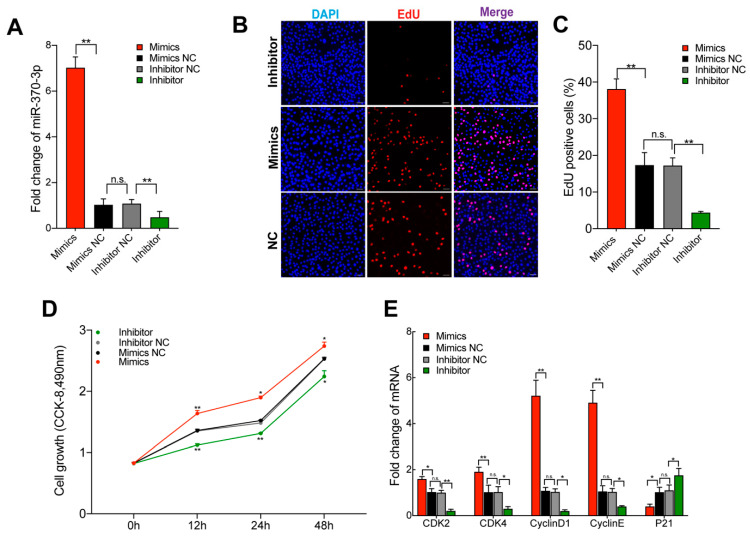
miR-370-3p inhibits preadipocyte proliferation. After 3T3-L1 cells were transfected with miR-370-3p mimics, inhibitors or Mimics NC and Inhibitor NC: (**A**) the transfection efficiency was measured using qRT-PCR; (**B**,**C**) EdU evaluated cells proliferation; (**D**) CCK-8 analysis; (**E**) the expression of genes associated with cell proliferation was measured by using qRT-PCR. Scale bars 50 μm. All results are presented as means ± SEM. n = 3 per treatment. Each treatment represents the mean of three replicates. * *p* < 0.05; ** *p* < 0.01, n.s. no significant difference.

To evaluate the role of miR-370-3p in 3T3-L1 adipocyte proliferation, we separately transfected the 3T3-L1 cell with miR-370-3p mimics, inhibitor, and negative control. As shown in Figure 2A, we successfully overexpressed or repressed the expression of miR-370-3p. We performed an EdU and CCK-8 cell proliferation assay to determine whether miR-370-3p affects cell proliferation. As shown in Figure 2B–D, the results showed that the EdU-positive cell ratio was upregulated after miR-370-3p and the opposite trend could be seen in the transfected miR-370-3p inhibitor group consisting of the CCK-8 assay results. We further examined the cell cycle and cell proliferation marker genes during cell proliferation after 3T3-L1 adipocytes were transfected. The qRT-PCR results showed that the expression of Cyclin-dependent kinase 2 (CDK2), Cyclin-dependent kinases 4 (CDK4), Cyclin D1 (CyclinD1), and Cyclin E1 (CyclinE) were significantly upregulated after miR-370-3p mimics were transfected, whereas the miR-370-3p inhibition group showed the opposite trend (Figure 2E). Moreover, after transfection, we further detected the expression levels of Cyclin-dependent kinase inhibitor 1 (p21), which used to be a cell-cycle-arrest regulator. The p21 expression was decreased after miR-370-3p overexpression. Therefore, the results suggested that miR-370-3p may promote adipocyte proliferation.

### 2.3. miR-370-3p Inhibits Preadipocyte Differentiation

**Figure 3:**
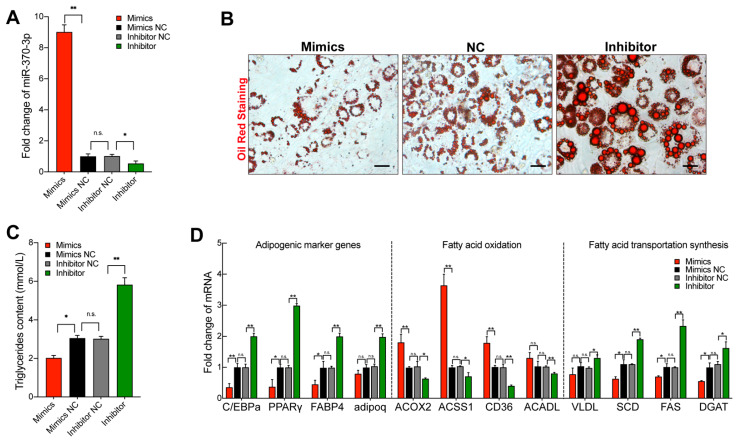
miR-370-3p inhibits preadipocyte differentiation. The transfection efficiency of transfection with miR-143-3p mimics or inhibitors in 3T3-L1 cells cultured in differentiation medium. (**A**)The transfection efficiency in 3T3-L1 cells of miR-370-3p. (**B**) Oil Red O staining, (**C**) triglycerides content, and (**D**) the expression of marker genes related to adipogenic, fatty acid oxidation, and fatty acid transportation synthesis. Scale bars 50 μm. All results are presented as means ± SEM. n = 3 per treatment. Each treatment represents the mean of three replicates. * *p* < 0.05; ** *p* < 0.01, n.s. no significant difference.

To evaluate the function of miR-370-3p in adipocyte differentiation, 3T3-L1 cells were transfected with miR-370-3p mimics or miR-370-3p inhibitor during the 12-day differentiation period. As shown in Figure 3A, the transfection efficiency was detected, and the expression of miR-370-3p was significantly increased after miR-370-3p mimics were transfected and decreased in response to miR-370-3p inhibition, which was transfected separately compared with the negative control group. As shown in Figure 3B, using oil red staining, we found that inhibiting miR-370-3p expression increased the formation of lipid droplets when compared with the negative control group, and the overexpression of the miR-370-3p group showed the opposite results. Unanimously, the triglyceride analysis showed that miR-370-3p overexpression represses the adipocyte triglycerides’ accumulation and the inhibition of miR-370-3p increased the triglyceride accumulation when compared with the negative control (NC) group (Figure 3C). To further confirm the above results, we examined the expression level of C/EBPα, PPARγ, FABP4, and Adiponectin, C1Q and collagen domain (adipoq). Consistently, we found that the transfection of miR-370-3p mimics or inhibitors significantly inhibited or enhanced these adipogenic genes. These results indicate that miR-370-3p may inhibit adipocyte differentiation (Figure 3D). In addition, we evaluated the expression level of genes that participate in fatty acid synthesis and fatty acid oxidation after cells were separately transfected with miR-370-3p mimics, inhibitor, and negative control. As anticipated, the expression of genes associated with fatty acid oxidation was promoted, and the expression of genes involved in fatty acid synthesis was repressed after miR-370-3p overexpression compared with the negative control group. Instead, the inhibition of miR-370-3p induced the opposite effects for fatty acid metabolism-related genes. The above results suggest that miR-370-3p promoted fatty acid oxidation but inhibited fatty acid synthesis during 3T3-L1 preadipocyte differentiation (Figure 3D). In summary, these results demonstrate that miR-370-3p negatively mediates 3T3-L1 preadipocyte differentiation and may be implicated in fatty acid metabolism.

### 2.4. miR-370-3p Changes Mature Adipocyte Fatty Acid Composition In Vitro and In Vivo, and Inhibit Adipogenesis In Vivo

**Figure 4:**
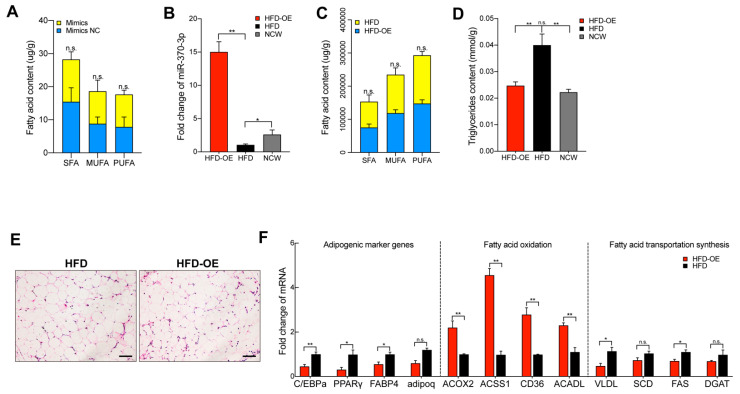
miR-370-3p regulates fatty acid composition both in vitro and in vivo. (**A**) Saturated fatty acids (SFA), monounsaturated fatty acids (MUFA), and polyunsaturated fatty acids (PUFA) content in mimic group and control group. (**B**) Measuring the expression of miR-370-3p in HFD-OE (high-fat-diet-induced obesity mice which overexpressed miR-370-3p), HFD- and NCW-fed mice. (**C**) SFA, MUFA, PUFA content in HFD and HFD-OE. (**D**) Triglyceride content. (**E**) H&E staining for adipose tissues from HFD and HFD-OE. (**F**) The mRNA levels of C/EBPa, PPARγ, FABP4, adipoq, ACOX2, ACSS1, CD36, ACADL, VLDL, SCD, FAS, and DGAT were measured by qRT-PCR. Scale bars 50 μm. All results are presented as means ± SEM. n = 3 per treatment. Each treatment represents the mean of three replicates. * *p* < 0.05; ** *p* < 0.01, n.s. no significant difference.

Fatty acids are important signaling molecules that form the core of adipose tissue components [14]. Thus, we examined whether the fatty acid composition of mature adipocytes changed with miR-370-3p. We measured the fatty acid composition after 3T3-L1 adipocyte-induced differentiation and transfected this with miR-370-3p mimics for eight days. As shown in Table 1, gas chromatography indicated that miR-370-3p overexpression significantly inhibited C14:0, C18:0 and had a positive influence on C15:1, C18:2n6c, C18:3n3, C20:3n3, C20:5n3, C20:4n6 and C22:6. In addition, C6:0, C8:0, C10:0, C12:0, C13:0, C15:0, C16:0, C16:1, C17:0, C18:1n9c, C20:0, C20:2 and C20:3n6 showed no significant difference between miR-370-3p mimics and the negative control group. Furthermore, the overexpression of miR-370-3p did not change the SFA, PUFA and MUFA contents compared with the negative control group (Figure 4A).

To further confirm the function of miR-370-3p in adipogenesis, we performed an in vivo experiment. Mice were injected with miR-370-3p overexpression vector. As shown in Figure 4B–F, the results were consistent with our in vitro results. The adipocyte area in epididymal fat was significantly reduced in the high-fat-diet-induced obesity mice, which overexpressed miR-370-3p (HFD-OE) group, and the TG content in eWAT also decreased. Following this, we detected the adipogenic, fatty acid oxidation, and fatty acid transportation synthesis marker genes. As expected, this tendency is consistent with the in vitro results. Furthermore, we also examined fatty acid composition in eWAT (Table 2). We found that C14:0 and C18:0 were also significantly decreased in the HFD-OE group, and the overexpression of miR-370-3p had a facilitating effect on C14:1, C15:1, C18:2n6c, C20:3n3, C20:5n3. The remaining fatty acid composition did not change significantly. In summary, these results showed that miR-370-3p regulates the adipogenic process and might change fatty acid composition during adipogenesis both in vitro and in vivo.

### 2.5. Mknk1 Is a Target Gene of miR-370-3p

**Figure 5:**
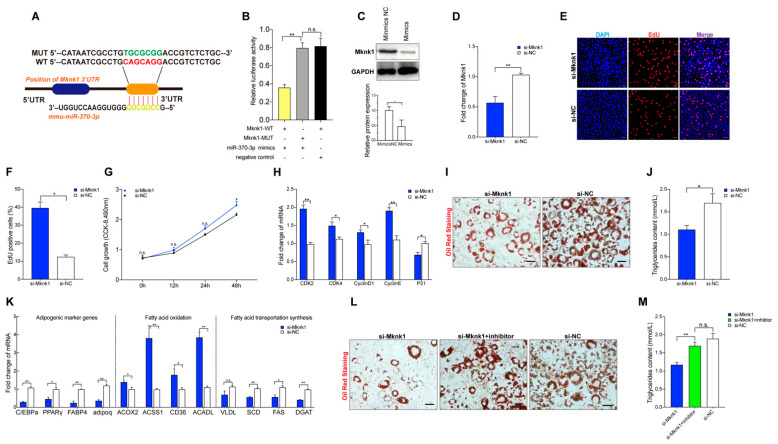
Mknk1 is a target gene of miR-370-3p. (**A**) Binding site and seed region of miR-370-3p are indicated in red. (**B**) Luciferase assays revealed the suppressive effect of miR-370-3p on the activity of Mknk1. (**C**) The protein expression level of Mknk1 48 h after transfection. (**D**) The expression levels of Mknk1 after cells were transfected with mimics, inhibitors, or NC. (**E**,**F**) EdU analysis, respectively. Moreover, (**G**) cell proliferation was evaluated at 0 h, 12 h, 24 h, and 48 h of proliferation by performing CCK-8. (**H**) qRT–PCR analysis of genes related to the cell cycle. (**I**) Cells were stained with oil red O. (**J**) Triglyceride content. (**K**) The expression levels of gene-related adipogenesis, fatty acid oxidation, and fatty acid transportation synthesis were measured by qRT-PCR. (**L**) Cells were stained with oil red O. (**M**) Triglyceride content. Scale bars 50 μm. All results are presented as means ± SEM. n = 3 per treatment. Each treatment represents the mean of three replicates. * *p* < 0.05; ** *p* < 0.01, n.s. no significant difference.

To explore the underlying mechanism of miR-370-3p inhibition of 3T3-L1 adipocyte differentiation, it is necessary to perform a bioinformatics analysis to predict the target genes of miR-370-3p (TasrgetScan, Miranda, miRDataBase). The results revealed that Mknk1 was predicted as a target gene of miR-370-3p. Interestingly, as shown in Figure 1C, the expression of Mknk1 was significantly increased in the HFD group, which showed the opposite trend to miR-370-3p. To explore whether Mknk1 is a direct target gene of miR-370-3p, we constructed a wild-type (WT) and mutant (MUT) luciferase reporter vector of the Mknk1 3’UTR region (Figure 5A). Moreover, the WT or MUT dual-luciferase vectors were co-transfected with miR-370-3p mimics into Hela cells. Dual-luciferase reporter assay results demonstrated that the fluorescence value was significantly decreased in the WT group compared with the MUT group and control group (Figure 5B). In addition, we also detected the protein level of MKNK1 after the miR-370-3p mimics’ transfection. As shown in Figure 5C, the expression level of MKNK1 was significantly downregulated in the miR-370-3p mimics group. Therefore, we performed a further functional validation of Mknk1. si-Mknk1 and si-NC were transfected into 3T3-L1 preadipocyte in proliferation medium or differentiation medium. As shown in Figure 5D–H, the inhibition of Mknk1 by siRNA significantly decreased the relative expression level of Mknk1 and CCK-8 detection. EdU proliferation assay results showed that the inhibition of Mknk1 had a significant, positive effect on 3T3-L1 preadipocyte proliferation. Moreover, this result was also confirmed by the qRT-PCR analysis of cell-cycle markers. Furthermore, Mknk1 inhibition caused the lipid droplet reduction detected by oil red stain assay (Figure 5I,J)). qRT-PCR analysis showed that si-Mknk1 caused the adipogenic genes to become downregulated, and this effect was reversed by miR-370-3p inhibitor transfection into 3T3-L1 adipocyte (Figure 5K–M)). Taken together, the findings suggest that miR-370-3p might regulate the proliferation and differentiation of preadipocytes by targeting Mknk1.

## 3. Discussion

White adipose tissue is a significant energy storage organ in mammals [15,16]. Furthermore, white adipose tissue is also an endocrine organ involved in the functioning of endocrine, autocrine and paracrine signaling. miR-370-3p is implicated in many critical biological processes such as cancer cell migration and invasion. Previous studies on miR-370-3p have focused on its role in cancer research [17,18]. For instance, miR-370-3p might be a crucial epigenetic biomarker of thyroid cancer [19]. In addition, miR-370-3p could inhibit tumor cell migration and invasion in various cancers, such as breast cancer [20]. However, the role of miR-370-3p in adipogenesis has not been investigated to date. In this work, our study showed that miR-370-3p decreased during preadipocyte differentiation. Moreover, the function of miR-370-3p was demonstrated by the preadipocyte proliferation and differentiation assay experiments, and we investigated the fatty acid composition of adipocyte after the transfection of miR-370-3p mimics. The direct target relationship between miR-370-3p and Mknk1 was validated by a dual-luciferase experiment. Based on our present study, we confirmed that miR-370-3p promotes preadipocyte proliferation and inhibits adipocyte differentiation by targeting Mknk1. Moreover, we found that miR-370-3p inhibition of adipocyte differentiation may be associated with the fatty acid composition, which is changed by the transfection of miR-370-3p mimics. Excessive intake of a high-fat-diet-induced obesity and the accumulation of triglycerides in serum and white adipose tissue [21,22]. We found that after high-fat-diet-induced mice obesity, TG and TC serum levels were upregulated, and the expression of adipogenesis-related genes was increased. The expression of miR-370-3p was downregulated in the eWAT of the HFD group, and Mknk1 expressed the opposite trend. Otherwise, 3T3 L1 cells are extensively used as a model for studying adipogenesis [23,24]. In this case, we tested the expression of miR-370-3p during 3T3-L1 cell differentiation. We found that, during 3T3-L1 differentiation, the expression of miR-370-3p was upregulated from 0 to 4 days and decreased from 4 to 8 days. These results indicate that miR-370-3p is involved in the biological process of adipogenesis. Adipocyte hyperplasia involves preadipocyte proliferation, which caused a subsequent differentiation referred to as adipogenesis [25]. Numerous studies have reported that, during preadipocyte proliferation and differentiation, related miRNAs play an essential role in this highly ordered and complex process [26,27,28]. As shown in our study, Mknk1 was sponged by miR-370-3p. Furthermore, based on our observation, miR-370-3p promotes preadipocyte proliferation and inhibits differentiation by repressing Mknk1. microRNA participates in the biological process through seed sequence matching [29]. MAP kinase-interacting kinases act as integration points for multiple biochemical signals and are involved in a variety of cellular processes, such as proliferation, differentiation, transcriptional regulation and development. Mknk1 is an MAP kinase-interacting kinases, predicted by the Targetscan program as an miR-370-3p target gene. Furthermore, the target sequence of miR-370-3p attached to the 3’UTR region of Mknk1 was highly conserved in human and mouse species based on sequence alignment. Meanwhile, the pre-experimental results of the dual luciferase assay showed that the miR-370-3p mimic significantly inhibited the luciferase activity of the reporter gene associated with the mouse Mknk1 3’ UTR, with no significant difference between the other groups. The involvement of Mknk1 in adipocyte differentiation was also improved in previous studies. MAP kinase-interacting kinases MNK1(Mknk1) is a target gene of miR-370-3p, which reported that the HFD-fed MNK1-knockout (KO) mice protected against diet-induced glucose intolerance and insulin resistance compared to HFD-fed wild-type animals [11]. To further determine whether the reduction in Mknk1 inhibits adipocyte lipid accumulation, we used siRNA against Mknk1 to simulate the role of miR-370-3p. The results suggested that si-Mknk1 transfection significantly decreased the lipid storage and adipogenesis-related genes’ expression level in 3T3-L1 adipocyte cells. These findings demonstrated that miR-370-3p might regulate obesity and related metabolic problems by modulating Mknk1.

Fatty acids are important components of adipose tissue and provide energy in mammals through β-oxidation. They also synthesize triglycerides as energy reserves in the body. In addition, triglycerides are a major component of fats, with each glycerol linking three units of fatty acids to form triglycerides, and different fatty acid triglyceride types have very different effects on lipid metabolism in the body. For example, the replacement of long-chain fatty acids by medium-chain fatty acids in the diet to form more medium-chain fatty acid triglycerides is effective in suppressing obesity, and the large accumulation of long-chain fatty acids in the body directly increases the risk of type 2 diabetes [30,31]. This evidence suggests that the fatty acid composition of adipocytes has a direct impact on lipid metabolism in mammals. Since the formation of different types of triglycerides is closely related to the fatty acid composition of adipose tissue, it is important to find the regulatory factors that can regulate the fatty acid composition of adipose tissue development to treat obesity and related metabolic diseases. The fatty acids are classified based on the number of double bonds between carbon atoms, with saturated fatty acids (SFAs), monounsaturated fatty acids (MUFAs), and polyunsaturated fatty acids (PUFAs) [32,33,34,35]. Furthermore, fatty acids play an essential role in lipid metabolism as they supply a variety of precursors. Our previous report showed that miR-125 could impair the composition of porcine intramuscular fat [36]. Moreover, a study indicated that SSC-miR-141 [37], which is downregulated in high backfat tissues that target FASN, might inhibit fatty acid synthesis in pig backfat tissues. Here, we showed that miR-370-3p inhibited 3T3-L1 cell adipogenesis and changed the fatty acid composition in mature adipocytes. In addition, we also examined the fatty acid composition of mice that overexpressed miR-370-3p. The results show that miR-370-3p also changed the fatty acid composition and repressed the eWAT differentiation in vivo. Thus, the evidence from this study showed that miR-370-3p affects adipocyte differentiation and changes fatty acid composition. Therefore, considering all these miR-370-3p factors and metabolic syndromes, miR-370-3p might play an important role in obesity and metabolic problems.

## 4. Conclusions

In the present study, the expression of miR-370-3p was found to be downregulated in the adipose tissues of HFD-fed mice and gradually decreased during 3T3-L1 preadipocyte differentiation. Functional analysis showed that miR-370-3p promoted 3T3-L1 preadipocyte proliferation and inhibited differentiation by directly targeting Mknk1. Furthermore, miR-370-3p was shown to upregulate the genes associated with fatty acid oxidation and downregulate genes involved in fatty acid synthesis. The above results indicated that the downregulation of miR-370-3p in adipose tissues was perfectly negatively correlated with adipogenesis in mice, and miR-370-3p might become a potential therapeutic target for obesity treatment.

## 5. Materials and Methods

### 5.1. Ethics Statement

The study was conducted under the approval of the Ethics Committee of Sichuan Agriculture University. All animal care and procedures performed in this study were conducted according to the Guidelines for Animal Experiments of Sichuan Agriculture University (Approval No.20210156).

### 5.2. Experimental Animals

All C57/BL6 mice were purchased from Dashuo company (Chengdu, China). Male C57/bl6 mice were fed a normal control diet or high-fat diet for six weeks. All mice were allowed free access to water and food, and both normal chow (NCW) and HFD group mice were kept under a 12 h dark–12 h light cycle. After sampling was completed, the serum levels of total cholesterol (TC) and triglycerides (TG) were measured. Moreover, the epididymal WAT (eWAT) was weighted to evaluate adipose tissue and stored at −80 °C for further analysis. In brief, mouse overexpression of the miR-370-3p model was found with miR-370-3p agomir.

### 5.3. Cell Culture and Transfection

Before transfection and differentiation, mouse 3T3-L1 preadipocyte and Hela cell were maintained in Dulbecco’s modification of Eagle’s medium (DMEM) containing 10% Fetal Bovine Serum (FBS) (proliferation medium, PM) at 37 °C with 5% CO_2_. To induce the 3T3-L1 preadipocytes cell differentiation, the preadipocytes were seeded on 6-well or 12-well plates and maintained with DMEM by fasting for two days. Cells were grown to confluence to maximize cell–cell contact for the purpose of lipid droplet formation. In addition, cells were cultured in a differentiation medium containing 10% FBS, 10 μg/mL insulin, one μM dexamethasone, and 0.5 mM IBMX, and the medium was changed to a maintenance media containing 10% FBS and 10 μg/mL insulin after two days, as described by our previous study [38].

In addition, the transfection of miR-370-3p mimics, inhibitor, negative control, and si-Mknk1,si-NC was performed to explore the effect of miR-370-3p and Mknk1 on 3T3-L1 preadipocyte proliferation and differentiation. Using lipo3000 (Invitrogen, Waltham, MA, USA) and opti-MEM, cells were transfected with miR-370-3p mimics, inhibitor, negative control, and si-Mknk1, according to the lipo3000 instructions.

### 5.4. Cell Proliferation Assay

Cell proliferation was found by 5-ethynyl-20-deoxyuridine (EdU) and CCK-8 assay (CCK-8, Zhuangmeng, Beijing, China). According to the instructions, the cells were seeded in 96-well plates and transfected with mimics, inhibitor, negative control, siRNA, and siRNA negative control. Finally, we performed absorbance detection at 450 nm to measure cell proliferation.

For the EdU assay, the cells were seeded in 96-well plates, and transfection was performed 12 h after plating the cells. As described in a previous study, the cells were maintained by proliferation medium 24 h, and 100 mL for 50 mM EdU were incubated per well. After staining, the plates were observed with a microscope (Nikon TE2000 microscope, Nikon, Tokyo, Japan).

### 5.5. Isolation of RNA and RT-PCR

According to the manufacturer’s instructions, the total RNA was isolated with RNAiso Plus (TaKaRa, Dalian, China). Furthermore, cDNA was made using a PrimeScript TM RT Reagent Kit with gDNA Eraser (TaKaRa, Dalian, China) and Mir-X miRNA First-Strand Synthesis Kit (TaKaRa, Dalian, China). Quantitative real-time PCR (qRT-PCR) reactions were performed as shown in a previous study [39]. The primer sequences used for qRT-PCR are listed in Supplementary Materials Table S1.

### 5.6. Oil Red O Staining and Triglyceride Assay

For Oil Red O staining, the cells were washed with PBS and fixed with cell fixing solution for 1 h. (Servicebio, Wuhan, China). After the cells were fixed, the cell samples were washed three times by PBS, incubated with 0.5% Oil Red O 10min, and the images were captured using an Olympus IX53 microscope (Olympus). Adipose tissue samples and cells were collected for Total Cholesterol and Triglyceride analysis, as described in the instructions (Nanjing Jiancheng Bioengineering Institute, China). The samples were disrupted to prepare a homogenate, then centrifuged at 2500 rpm/min for 10 min. Finally, the supernatant was collected and tested. The relevant OD value was determined following the instructions of the test kit.

### 5.7. Luciferase Reporter Assay

The wild-type 3′UTR of Mknk1(WT-Mknk1), mutant-type 3’UTR of Mknk1(MUT-Mknk1) were cloned into the psi-CHECKTM-2 vector, and the vectors were constructed by TsingKe company (TsingKe Biotech, Chengdu, China). For further dual-luciferase analysis, the abovementioned vectors were co-transfected with miR-370-3p mimics, mimics NC, and negative control, respectively, into Hela cells with lipo3000. Cells were starved without serum for 48 h, and luciferase activities were measured with the Dual-Glo Luciferase Assay System (Promega, Madison, WI, USA) according to the kit instructions.

### 5.8. Westen Blotting

In brief, we used a Lysis buffer to extract the 3T3-L1 cell protein, which was electrophoresed on SDS-PAGE and transferred to a 0.45 μm PVDF membrane (Bio-Rad). As described in our previous study [39], after the transfection, we used 5% nonfat milk to block each membrane for about 2 h. Then, the membrane was incubated with the primary antibodies (Mnk1(A-4):sc-133107,SANTA CRUZ BIOTECHNOLOGY, INC) at 4°C overnight, after being washed three times by TBST buffer. Finally, we detected the bands using ECL (BioSharp, Hefei, China) after secondary antibody incubation.

### 5.9. Statistical Analysis

All data are expressed as means ± SEM, and statistical analyses were performed using Prism 9.0 software. Differences between groups were analyzed by applying the Student’s two-tailed *t*-test for two parametric groups and one-way analysis for the three least parametric groups. A value of *p*-value < 0.05 indicated a significant difference, * *p* < 0.05, ** *p* < 0.01.

## Figures and Tables

**Table 1:** miR-370-3p regulates fatty acid composition in vivo.

Fatty Acid	NC	Mimics	Significance
*C6:0*	0.29	0.28	Increase ^NS^
*C8:0*	0.1	0.11	Increase ^NS^
*C10:0*	0.16	0.16	Increase ^NS^
*C11:0*	0	0	——
*C12:0*	0.26	0.28	Increase ^NS^
*C13:0*	0.26	0.25	Increase ^NS^
*C14:0*	1.6	1.35	Decrease *
*C14:1*	0	0	——
*C15:0*	0.38	0.4	Increase ^NS^
*C15:1*	0.87	0.95	Increase *
*C16:0*	4.01	3.64	Decrease ^NS^
*C16:1*	0.24	0.22	Decrease ^NS^
*C17:0*	0.44	0.45	Increase ^NS^
*C17:1*	0	0	——
*C18:0*	6.59	4.77	Decrease **
*C18:1n9t*	0	0	——
*C18:1n9c*	7.72	8.63	Increase ^NS^
*C18:2n6t*	0	0	——
*C18:2n6c*	1.39	1.63	Increase *
*C18:3n6*	0	0	——
*C18:3n3*	0.58	0.67	Increase *
*C20:0*	0.62	0.48	Decrease ^NS^
*C20:1*	0	0	——
*C20:2*	0.59	0.61	Increase ^NS^
*C21:0*	0	0	——
*C20:3n6*	1.22	1.54	Increase ^NS^
*C20:4n6*	2.18	2.45	Increase *
*C20:3n3*	0.57	0.63	Increase **
*C20:5n3*	0.49	0.89	Increase **
*C22:0*	0	0	——
*C22:1n9*	0	0	——
*C22:2n6*	0	0	——
*C23:0*	0.12	0.13	
*C24:0*	0	0	——
*C24:1*	0	0	——
*C22:6*	1.42	1.97	Increase *

* *p* < 0.05; ** *p* < 0.01, NS means no significant difference.

**Table 2:** miR-370-3p regulates fatty acid composition in vitro.

Fatty Acid	HFD-NC	HFD-OE	Significance
*C6:0*	29.4	36.17	Increase ^NS^
*C8:0*	10.23	13.32	Increase ^NS^
*C10:0*	50.51	61.86	Increase ^NS^
*C11:0*	1	1.11	Increase ^NS^
*C12:0*	263.84	334.95	Increase ^NS^
*C13:0*	6.2	6.17	Increase ^NS^
*C14:0*	5937.03	5544.6	Decrease **
*C14:1*	258.6	305.88	Increase **
*C15:0*	329.27	355.14	Increase ^NS^
*C15:1*	2.87	4.44	Increase **
*C16:0*	48,897.32	47,001.13	Decrease ^NS^
*C16:1*	19,479.61	21,348.67	Increase ^NS^
*C17:0*	586.42	588.27	Increase ^NS^
*C17:1*	767.72	832.1	Increase ^NS^
*C18:0*	21,122.84	21,110.67	Decrease **
*C18:1n9t*	0	0	——
*C18:1n9c*	92,141.78	92,929.21	Increase **
*C18:2n6t*	0	0	——
*C18:2n6c*	43,726.38	44,619.86	Increase **
*C18:3n6*	0	0	——
*C18:3n3*	3682.38	4043	Increase ^NS^
*C20:0*	469.59	446.31	Decrease ^NS^
*C20:1*	3283.17	3103.03	Decrease ^NS^
*C20:2*	1369.2	1390.44	Increase ^NS^
*C21:0*	0	0	——
*C20:3n6*	507.39	539.02	Increase ^NS^
*C20:4n6*	801.83	778.48	Decrease ^NS^
*C20:3n3*	1899.48	2096.02	Increase **
*C20:5n3*	173.99	184.22	Increase **
*C22:0*	72.72	69.17	Decrease **
*C22:1n9*	149.16	137.78	Decrease ^NS^
*C22:2n6*	11.57	12.34	Increase ^NS^
*C23:0*	12.71	13.08	Increase ^NS^
*C24:0*	52.09	51.81	Decrease ^NS^
*C24:1*	88.6	85.59	Decrease ^NS^
*C22:6*	1157.08	1157.85	Increase ^NS^

** *p* < 0.01, NS means no significant difference.

## Data Availability

The data presented in this study are available on request from the corresponding author.

## Supplementary Materials

The following are available online. Table S1. The primer sequences used for qRT-PCR.

## Abbreviations

*PPARγ*	*Peroxisome proliferator activated receptor γ*
*C/EBPα*	*CCAAT/enhancer binding protein α*
*FABP4*	*Adipocyte fatty acid-binding protein 4*
*CDK2*	*Cyclin-dependent kinase 2*
*CDK4*	*Cyclin-dependent kinases 4*
*CyclinD1*	*Cyclin D1*
*CyclinE*	*Cyclin E1*
*P21*	*Cyclin-dependent kinase inhibitor 1*
*Adipoq*	*Adiponectin, C1Q and collagen domain containing*
*ACOX2*	*Acyl-Coenzyme A oxidase 2, branched chain*
*ACSS1*	*Acyl-CoA synthetase short-chain family member 1*
*CD36*	*CD36 molecule*
*ACADL*	*Acyl-Coenzyme A dehydrogenase, long-chain*
*VLDL*	*CD320 antigen*
*SCD*	*Stearoyl-Coenzyme A desaturase 1*
*FAS*	*Fatty acid synthase*
*DGAT*	*Diacylglycerol O-acyltransferase 1*
*PNPLA3*	*Patatin-like phospholipase domain containing 3*
*BMP2*	*Bone morphogenetic protein 2*
*Mknk1*	*MAP kinase-interacting serine/threonine kinase 1*
miRNA	MicroRNA
UTR	3′untranslated region
RISC	RNA-induced silencing complex
HFD	high fat diet
NCW	normal chow
eWAT	epididymal white adipose tissue
iWAT	inguinal white adipose tissue
TG	triglycerides
TC	total cholesterol
NC	negative control
HFD-OE group	high fat diet-induced obesity mice which overexpressed miR-370-3p
WT	wild type
MUT	mutant
SFAs	saturated fatty acids
MUFAs	monounsaturated fatty acids
PUFAs	polyunsaturated fatty acids
EdU	5-ethynyl-20-deoxyuridine
qRT-PCR	Quantitative real-time PCR
WT-Mknk1	wild-type 3′UTR of Mknk1
MUT-Mknk1	mutant-type 3’UTR of Mknk1
