# Supplementary material for: miR-370-3p Regulates Adipogenesis through Targeting Mknk1
Source: Molecules. 2021 Nov 17;26(22):6926. doi: 10.3390/molecules26226926 (PMC8619113; doi:10.3390/molecules26226926)
Supplement: Supplementary file 1 [file molecules-26-06926-s001.zip › molecules-1419678-supplementary.pdf]

**Supplementary Table S1.** The primer sequences used for qRT-PCR. F: forward, R: reverse. U6 and *β-actin* were used as endogenous control genes for miRNA and mRNA, respectively.

| Gene | Primer sequence (5`→3`) |
|---|---|
| *Cyclin D1* | F: GTTGCTGGAATTTTCGGGGT<br>R: AGCGTCCCTGTCTTCTTTCA |
| *C/EBPα* | F:CAAGAACAGCAACGAGTACCG<br>R:GTCACTGGTCAACTCCAGCAC |
| *PPARr* | F:CTCCAAGAATACCAAAGTGCGA<br>R:GCCTGATGCTTTATCCCCACA |
| *CDK4* | F: GTCAGTTTCTAAGCGGCCTG<br>R: CACGGGTGTTGCGTATGTAG |
| *CDK2* | F: CCCTTCCCAAAGCCCTTTTC<br>R: GAAGAGGGGAAGAAGCTGGT |
| *Cyclin E* | F: AGCCTCGGAAAATCAGACCA<br>R: TCCTGTGCCAAGTAGAACGT |
| *p21* | F: GATGGCTTCGACACCATTCC<br>R:AGACGACACAGGTGAGGAAG |
| *FABP4* | F: TTTCCTTCAAACTGGGCGTG<br>R: CATTCCACCACCAGCTTGTC |
| *adipoq* | F: TGTTCCTCTTAATCCTGCCCA<br>R: CCAACCTGCACAAGTTCCCTT |
| *ACOX2* | F: AACCCAGGGGATCGAGTGT<br>R: CGCAGCTCAGTGTTTGGGAT |
| *ACSS1* | F: GTTTGGGACACTCCTTACCATAC<br>R: AGGCAGTTGACAGACACATTC |
| *CD36* | F: ATGGGCTGTGATCGGAACTG<br>R: GTCTTCCCAATAAGCATGTCTCC |
| *ACADL* | F: TCTTTTCCTCGGAGCATGACA<br>R: GACCTCTCTACTCACTTCTCCAG |
| *VLDL* | F: GGTCCAAGTCTCCGGCTCTA<br>R: AGCACATGACTCAATCCTACAGT |
| *SCD* | F:TTCTTGCGATACACTCTGGTGC<br>R:CGGGATTGAATGTTCTTGTCGT |
| *FAS* | F:TATCAAGGAGGCCCATTTTGC<br>R:TGTTTCCACTTCTAAACCATGCT |
| *Mknk1* | F: TAGTGAGCCTGTGTGTCCAG<br>R: CTGCGCTTCTCTTCTCGTTC |
| *DGAT* | F:CTGATCCTGAGTAATGCAAGGTT |

| | |
|---|---|
| | R:TGGATGCAATAATCACGCATGG |
| *miR-370-3p* | GCCUGCUGGGGUGGAACCUGGU |
| *U6* | F:CTCGCTTCGGCAGCACA |
| | R:AACGCTTCACGAATTTGCGT |
| *β-actin* | F:TGGAATCCTGTGGCATC CATGAAAC |
| | R:TAAAACGCAGCTCAG TAACAGTCCG |
